# Synthesis of Novel Triazoles, Tetrazine, Thiadiazoles and Their Biological Activities

**DOI:** 10.3390/molecules20022591

**Published:** 2015-02-02

**Authors:** Mohammed A. Al-Omair, Abdelwahed R. Sayed, Magdy M. Youssef

**Affiliations:** 1 Department of Chemistry, Faculty of Science, King Faisal University, Hofuf 31982, Saudi Arabia; E-Mails: alomair@kfu.edu.sa (M.A.A.-O.); mmm_youssef@yahoo.com (M.M.Y.); 2 Department of Chemistry, Faculty of Science, University of Beni Suef, Beni Suef 62511, Egypt; 3 Department of Chemistry, Faculty of Science, Mansoura University, Mansoura 35516, Egypt

**Keywords:** triazoles, tetrazine, thiadiazoles, antibacterial, antioxidant, DNA

## Abstract

An expedient synthesis of novel triazoles, tetrazine and thiadiazoles, using conveniently accessible and commercially available starting materials has been achieved. The synthesized compounds were characterized by spectroscopic and elemental analyses, and screened for their antibacterial activities against four different strains, namely *E. coli*, *P. aeruginosa*, *S. aureus* and *B. megaterium*. In particular, the compounds **5**, **24** and **26h** exhibited excellent antibacterial activities compared to the reference antibiotic. To get further insight about their behavior, these compounds were tested for their antioxidant activities via SOD-like activity, DPPH free radical scavenging activity, ABST and NO, which showed promising results. Furthermore, these compounds effectively promoted the cleavage of genomic DNA as well, in the absence of any external additives.

## 1. Introduction

In recent years, heterocyclic compounds have received considerable attention due to their significant importance in pharmacological and agricultural fields [1,2,3,4,5,6,7]. Notably, nitrogen-containing heterocycles exhibit excellent biological activities [8]. Free radicals and reactive oxygen species such as hydroxyl radical, hydrogen peroxide and superoxide radical anion are frequently synthesized through many biological processes and may be considered as indicators of biological inadequacy. Reactive oxygen species are also capable of damaging essential biomolecules such as nucleic acids, proteins, lipids, and carbohydrates and may cause DNA damage that can lead to mutations. Biological systems use antioxidant molecules such as nitrogen-containing heterocycles to neutralize the excessive levels of reactive oxygen and nitrogen species [9].

1,2,4-Triazole rings are typically planar 6π-electron aromatic systems and extensive research has been carried out in this domain [10,11]. These represent one of the most bioactive classes of compounds, which display diverse biological activities in the medicinal and agrochemical fields, including anti-inflammatory [12,13], antifungal [14,15], herbicidal [16], antimicrobial [16,17], antiparasitic [18], cytostatic [19], and brassinosteroid biosynthesis inhibitory activities [20]. Triazolopyrimidines possess a range of biological activities, including activities against *Aspergillus* and *Pencicillium* species [21,22,23,24,25,26], and have been evaluated as bioregulator agents [27]. Tetrazines are of considerable interest not only because of their inherent biological potential [28], but also because of their value as building blocks in synthetic transformations. 1,2,3-Thiadiazoles have also been reported to have significant agricultural applications [29,30] and antiviral activities [31].

Hydrazones are of great interest to researchers because of their diverse biological and clinical applications. They have been reported to exhibit antimicrobial, anticonvulsant, analgesic, anti-inflammatory, antiplatelet, antitubercular and antitumor activities [32,33,34]. Small heterocyclic molecules that interact with DNA through recognition, binding, crosslinking or cleaving have gained significant attention and are considered as promising area of research in the fields of chemistry, biology and medicine. Such heterocyclic compounds are capable of hydrolyzing and manipulating DNA, and potentially can be used as chemotherapeutic agents [35]. In the present study, our synthetic investigations towards novel heterocyclic compounds, their biological evaluations and genomic DNA degradation activities are described.

## 2. Results and Discussion

### 2.1. Chemistry

**Scheme 1:**
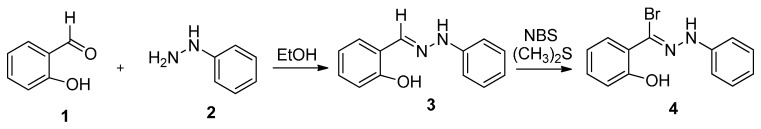
Synthesis of 2-hydroxy-*N*-phenylbenzohydrazonoyl bromide **4**.

In continuation of our active research in an area of hydrazonoyl halides and their reactions [36,37,38,39,40], a novel compound **4** was prepared by the reaction of salicylaldehyde (**1**) with phenylhydrazine (**2**) in boiling ethanol to afford salicylaldehydephenylhydrazone (**3**, Scheme 1) [41]. The hydrazone **3** was then treated with *N*-bromosuccinimide to give the desired 2-hydroxy-*N*-phenylbenzohydrazonoyl bromide (**4**). Spectroscopic data and microanalytical analyses confirmed the structure of bromide **4**.

**Scheme 2:**
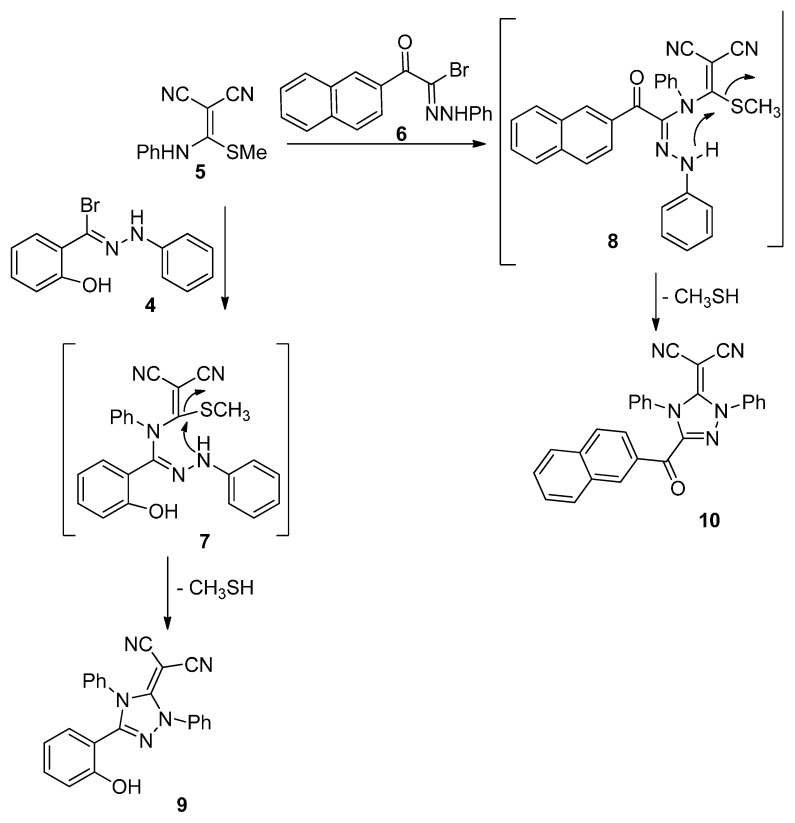
Synthesis of triazoline derivatives **9** and **10**.

Pleasingly, treatment of hydrazonoyl bromide **4** or 2-(2-phenylhydrazono)-2-bromo-1-(naphthalene-2-yl)ethanone (**6**) with 2-cyano-3-methylthio-3-phenylaminoacrylonitrile (**5**) in the presence of trimethylamine under reflux conditions proceeded smoothly to afford 1,2,4-triazole derivatives **9** and **10**, respectively (Scheme 2). It is proposed that the reactions involve the loss of hydrogen bromide and methanethiol during the course of reaction to provide the desired products **9** and **10**. The compounds were characterized by elemental analysis, spectral data (IR, MS, ^1^H-NMR spectra), which confirmed the absence of sulfur atoms.

**Scheme 3:**
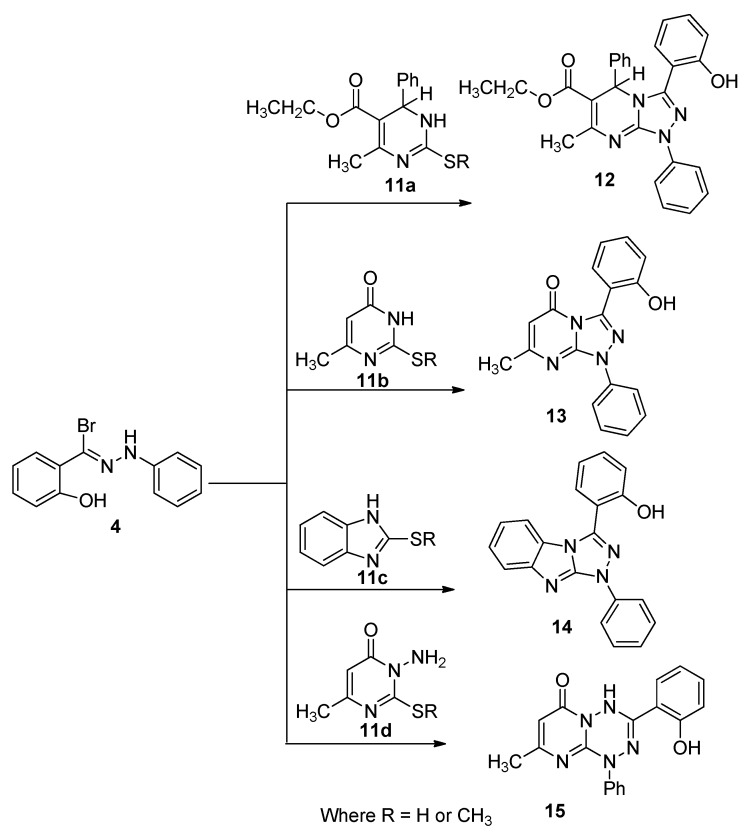
Synthesis of triazolines **12**–**14** and tetrazine **15**.

After successful accomplishment of this reaction, a series of novel compounds **12**–**15** were prepared via the reaction of hydrazonoyl bromide **4** with 2-thioxo or 2-methylthio derivatives **11a**–**d** in boiling chloroform, in the presence of triethylamine as a base catalyst until hydrogen sulfide or methanethiol gas ceased to evolve (Scheme 3). After standard work-up, the crude reaction mixture showed single spots on TLC for each reaction mixrture, from which the desired products **12**–**15** were isolated in excellent yields and characterized.

**Scheme 4:**
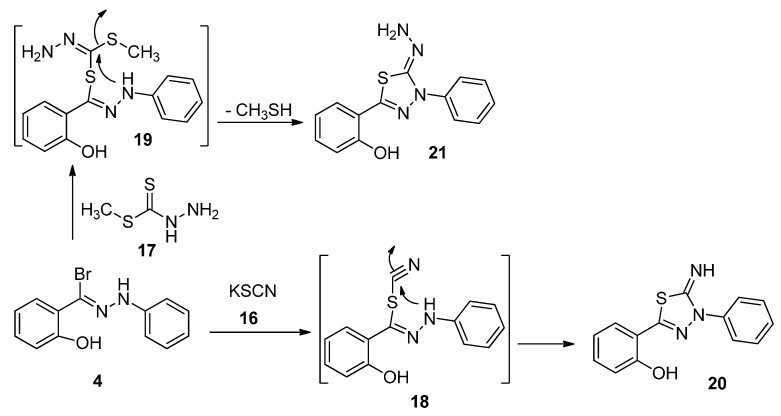
Synthesis of thiadiazoles **20** and **21**.

Treatment of hydrazonoyl bromide **4** with potassium thiocyanate (**16**) in hot ethanol provided 3-phenyl-5-(2-hydroxyphenyl)-1,3,4-thiadiazol-2(3*H*) imine (**20**) as sole isolated product via elimination of hydrogen bromide, followed by cyclization (Scheme 4). In similar manner, the reaction of hydrazonoyl bromide **4** with methyl hydrazinecarbodithioate (**17**) afforded the desired product **21** via elimination of hydrogen bromide and methanethiol.

**Scheme 5:**
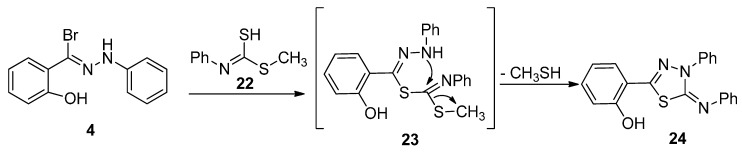
Synthesis of thiadiazole **24**.

Also, the reaction of hydrazonoyl bromide **4** with methyl hydrogenphenylcarbonimidodithioate (**22**) in EtOH/TEA gave thiadiazole **24** (Scheme 5), which was characterized by its elemental analysis and spectral data.

**Scheme 6:**
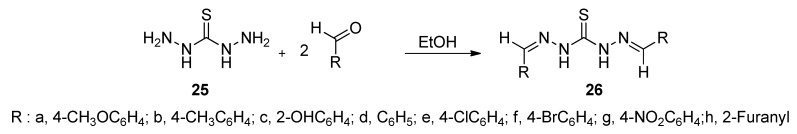
Synthesis of dihydrazones **26**.

In addition to this, 1,5-diarylidenethiocarbonohydrazide derivatives **26a**–**h** were prepared according to the literature methods [42,43,44], which involve the treatment of thiocarbohydrazide **25** with aldehydes in hot ethanol to afford the desired dihydrazones **26a**–**h** (Scheme 6).

### 2.2. Biology

#### 2.2.1. Antibacterial Activities

The antibacterial activity of the synthesized heterocyclic triazoles, tetrazine and thiadiazoles compounds were tested against both Gram-negative and Gram-positive bacteria using their single concentration in DMSO as a control. The inhibition zone against the growth of the verified bacteria for the compounds is given in Table 1. From the results, it is evident that compounds **5**, **24** and **26h** showed excellent antibacterial activity towards all the investigated bacterial strains compared to the tetracycline used as a reference antibiotic. The other compounds **4**, **14**, **26a**, **26b**, **26f** and **26c** revealed good antimicrobial activity towards the examined bacteria. The compounds **9**, **12**, **15**, **20**, **21**, **26d** and **26e** displayed moderate levels of antimicrobial activity, while compound **26g** offered the lowest antibacterial activity. From the antibacterial assay data presented in Table 1, it seems that enhanced biological activity for the compound **24** is due to its electron donating group (hydroxyl) and the poly-conjugated nature of the compound. For compound **26h**, the furan motif acts as an electron donating group as well as a diene in the Diels-Alder reaction and delocalization of electrons through the furan ring facilitates the electron density on the hydrazone structure [45]. The *ortho*-, *meta*- or *para*-position of the electron donating groups in the aromatic system plays a significant role in the bioactivities. In addition to this, the presence of heteroatoms such as oxygen, sulfur, and nitrogen also play a vital role in the observed antibacterial activity. It is also suggested that the sulfur-containing compounds might inhibit enzyme synthesis, since enzymes need specific groups for their activity and are especially susceptible to deactivation by the compounds. The presence of sulfur and nitrogen atoms in the structure of the active compounds facilitates their diffusion through the lipid layer of the microorganism membranes to the site of action, eventually killing them by linking with essential groups of certain cell enzymes [46].

**Table 1:** Effect of compounds on Gram-negative and Gram-positive microorganisms. Results expressed as inhibition zone diameter in mm.

Compound No.	Gram-Negative		Gram-Positive	
	*E. coli*	*P. aeruginosa*	*S. aureus*	*B. megaterium*
**4**	15	13	16	18
**5**	17	14	19	20
**9**	13	12	14	15
**12**	12	11	13	14
**14**	14	13	15	16
**15**	12	11	14	14
**20**	13	11	14	16
**21**	12	12	13	15
**24**	18	16	18	21
**26a**	13	12	13	15
**26b**	12	14	12	16
**26c**	13	14	12	17
**26d**	12	11	13	14
**26e**	13	12	14	13
**26f**	14	13	15	17
**26g**	9	11	11	10
**26h**	21	20	20	21
**Tetracycline**	20	18	22	24

#### 2.2.2. Minimum Inhibitory Concentrations

The minimum concentration at which no growth was detected was taken as the MIC value. The MIC of the compounds against Gram-negative and Gram-positive bacterial strains was evaluated. Comparison of the MICs (in μg/mL) of the synthesized compounds and tetracycline used as a standard drug against susceptible organisms is presented in Table 2. It was found that compounds **4**, **5**, **14**, **24** and **26h** showed the highest inhibitory activity against all measured strains (MIC values in the range of 15–35 μg/mL). Compounds **9**, **12**, **15**, **20**, **21**, **26a**, **26b**, **26c**, **26f** and **26d** had good inhibitory activity against all tested strains (MIC range: 30–55 μg/mL). A plausible explanation for these results is that the antibacterial activity of compounds may result from the basic skeleton of the molecules as well as from the nature of the nitrogen and sulfur atoms and the presence of substituents such as NO_2_, Br, Cl, OH, CH_3_ and OCH_3_ groups.

**Table 2:** Minimum inhibitory concentration (μg/mL) of compounds against Gram-negative and Gram-positive microorganisms.

Compound No.	Gram-Negative		Gram-Positive	
	*E. coli*	*P. aeruginosa*	*S. aureus*	*B. megaterium*
**4**	25	30	30	35
**5**	15	15	20	20
**9**	35	40	35	45
**12**	40	45	50	50
**14**	20	30	35	35
**15**	45	40	45	50
**20**	40	45	50	40
**21**	35	40	50	45
**24**	15	20	20	20
**26a**	30	35	35	35
**26b**	30	30	35	35
**26c**	25	35	40	35
**26d**	40	45	45	55
**26e**	45	45	55	50
**26f**	35	40	40	35
**26g**	55	50	70	50
**26h**	15	20	20	20
**Tetracycline**	10	15	15	15

#### 2.2.3. DNA Cleavage Assay

**Figure 1:**
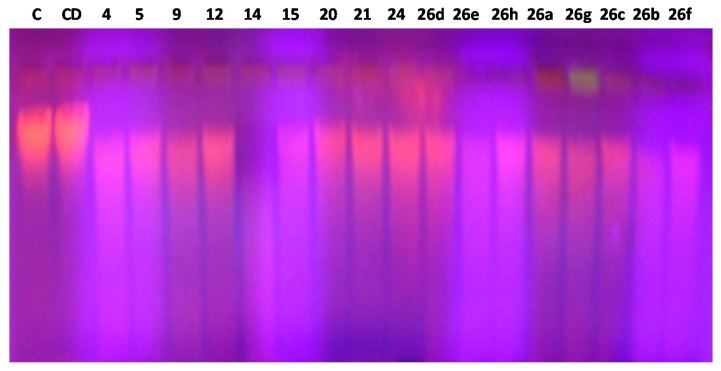
A figure showing the degradation effect of 4 μg of series (Lanes 3–19) on the genomic DNA isolated from *E. coli*, Lane1 *E. coli* DNA and lane 2 *E. coli* DNA + DMSO.

The ability of the compounds to cleave genomic DNA was studied and compared to the controls using the agarose gel electrophoresis technique. In the current study, the compounds displayed DNA degradation effect which proves their binding aptitude to the genomic DNA (Figure 1). When DNA was allowed to interact with compounds at 4 μg, DNA cleavage was noticed, as shown in Figure 1. The result indicates that the compounds **5**, **24** and **26h** are able to perform an efficient cleavage of DNA at 4 μg. Furthermore, the compounds **4**, **14**, **26a**, **26b**, **26f** and **26c** also demonstrated an efficient cleavage of the genomic DNA as demonstrated by agarose gel electrophoresis. The investigational observations revealed that the compounds have promising capability to degrade the genomic DNA *in vitro*. The obtained results also indicate that the examined compounds at 4 μg can effectively digest DNA. The weak DNA cleavage ability of the compounds **12**, **15**, **21**, **26d**, **26e** and **26g** may be attributed to the difference in the pharmacokinetic properties of the substituents in these compounds. These results suggest that the DNA cleavage activity depends on the particular structures of the studied compounds. It was observed that replacing one or more of the electron donating groups such as hydroxyl or furan ring results in an increase in the DNA cleavage affinity. The compounds thus may have use as endonuclease mimics due to their interesting structural features. The present study demonstrates that the compounds **5**, **24** and **26h** have a significant nuclease activity toward the cleavage of genomic DNA in the absence of any external additives. The DNA-binding evaluations are important for the rational strategy and construction of new and extra-efficient drugs targeting DNA. The DNA cleavage activity without any additive is an appreciated feature for these compounds, which provides a potential application of these compounds as chemotherapeutic agents in anticancer treatments.

#### 2.2.4. Superoxide Dismutase Mimetic Catalytic Activity

As some of the synthesized compounds demonstrated good DNA binding affinity, we envisaged studying their antioxidant activity too. Superoxide anions (O_2_^•−^) are originators for dynamic free radicals that have the potential to react with biological macromolecules thus inducing cell damage. Superoxide anions have a short half-life and consequently, these are being constantly produced. In this colorimetric based assay, the studies of O_2_^•−^ dismutation were accomplished by a catalyst and PMS as O_2_^•−^ photogenerator at room temperature. Inhibition of the reduction of NBT to formazan by the synthesized compounds was employed for recognition of the SOD-mimetic catalytic activity of these compounds in a phosphate buffer comparable to a biological environment. As the reaction proceeds, the formazan color is established and then it changes from colorless to blue, which is related with an increase in the absorbance at 560 nm. SOD decreases the superoxide ion concentration and thereby lowers the rate of formazan formation. In the SOD-like activity test, the compounds compete with NBT for oxidation of the generated superoxide ions. The more efficient the compound, the lower the concentration that corresponds to 50% inhibition of NBT reduction; this concentration is called the IC_50_. The data in Table 3 present the scavenging efficiency of each compound, giving its final concentration that produces efficient quenching of the superoxide anion radical. The compounds **26h**, **5**, **14** and **24** displayed a significant SOD-like activity and caused percent inhibitions of 53.3, 51.8, 50.4 and 50.2, respectively. In addition to this, compounds **26f**, **4**, **26a**, **26c**, **26b** and **9** exhibited a moderate SOD-like activity with the inhibition percentages of 46.4, 45.8, 44.3, 43.8, 42.7 and 40.5, respectively. However, the compounds **20**, **21**, **26e**, **12**, **15**, **26d** and **26g** displayed a weak SOD-like activity with inhibition percentages of 39.4, 38.5, 34.7, 33.6, 30.5, 30.3, and 29.6, respectively.

**Table 3:** Superoxide dismutase mimetic catalytic activity of compounds as antioxidant enzyme.

Compound No.	Δ through 4 min	% Inhibition
**Control**	0.548	—
**Horseradish peroxidase**	0.169	69.2
**4**	0.297	45.8
**5**	0.264	51.8
**9**	0.326	40.5
**12**	0.364	33.6
**14**	0.272	50.4
**15**	0.381	30.5
**20**	0.332	39.4
**21**	0.337	38.5
**24**	0.273	50.2
**26a**	0.305	44.3
**26b**	0.314	42.7
**26c**	0.308	43.8
**26d**	0.382	30.3
**26e**	0.358	34.7
**26f**	0.294	46.4
**26g**	0.386	29.6
**26h**	0.256	53.3

#### 2.2.5. DPPH Free Radical Scavenging Activity

**Figure 2:**
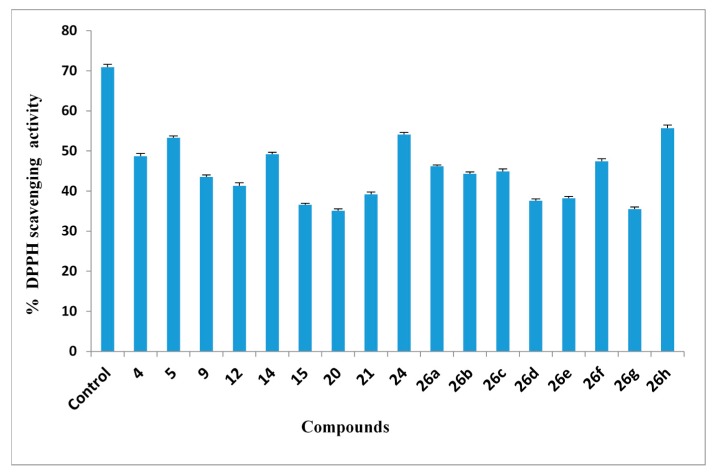
Antioxidant activities of compounds using DPPH.

The DPPH^•^ radical scavenging activity (RSA) evaluation is a typical assay used in antioxidant activity studies that offers a rapid technique for screening the RSA of specific compounds. The interaction of synthesized compounds with stable DPPH free radical indicates their free radical scavenging ability. The compounds showed antiradical activity by inhibiting DPPH radical (Figure 2). Majority of the tested compounds in the series revealed high to moderate interactions with the DPPH radical at 2 μg/mL concentration. The maximum antioxidant activity was observed in compounds in the following order **26h** > **24** > **5**, which displayed more than 50% inhibition, which is comparable to that of the standard (vitamin C) at a similar concentration, as shown in Figure 2. The presence of either electron-withdrawing or electron-donating groups in the aromatic rings may play a functional role in the activity. When the electron-donating groups such as furan ring is introduced in the compounds, the antioxidant activity is increasedm as reflected by compound **26h**, but electron-withdrawing groups such as NO_2_ or Cl decrease the activity, as observed for **26g** or **26e**, respectively. The other compounds exhibited moderate RSA in the order **14** > **4** > **26f** > **26a** > **26c** > **9** > **26b** > **12** (Figure 2). The antioxidant activity of these compounds is related with their electron delocalization or hydrogen radical donating ability to DPPH radical.

#### 2.2.6. ABTS Radical Cation Decolorization Assay

**Figure 3:**
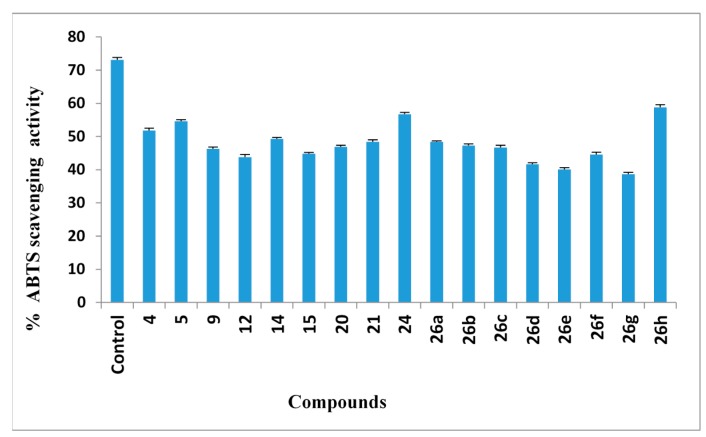
Antioxidant activities of compounds using ABTS.

The antioxidant activity evaluation employed here is one of the numerous assays that depends on determining the consumption of stable free radicals and used to assess the free radical scavenging activity of the examined compound. The reduction in colour intensity of the free radical solution due to scavenging of the free radical by the antioxidant material is determined colorimetrically at 734 nm. The test employs the radical cation formed from ABTS as a stable free radical. The benefit of the ABTS-derived free radical process over other approaches is that the produced colour remains stable for more than 1 h and the reaction is stoichiometric. The antioxidant activities of seventeen newly synthesized compounds were subjected to ABTS assessment (Figure 3). From these results, it is concluded that compounds **26h** > **24** > **5** > **4** exhibited more than 50% inhibition of the ABTS radical cation. On the other hand, the compounds **14** > **21** > **26a** > **26b** > **20** > **26c** > **26f** > **9** > **15** showed weak scavenging activities with lower than 50% inhibition of the ABTS (Figure 3).

#### 2.2.7. Nitric Oxide (NO) Scavenging Assay

**Figure 4:**
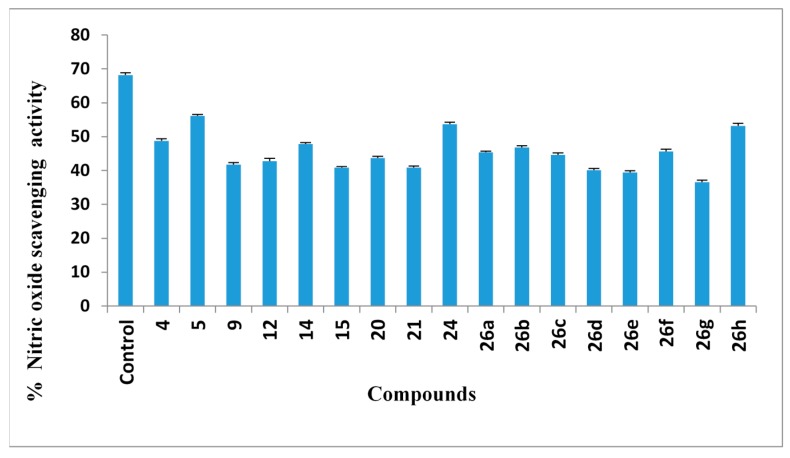
Antioxidant activities of compounds using NO.

The compounds were also examined for antioxidant properties by the nitric oxide scavenging activity assay (Figure 4). The compounds displayed nitric oxide radical scavenging activity in the following order: **5** > **24** > **26h** > **4** > **14** > **26b** > **26f** > **26a**. The other evaluted compounds **26c**, **21**, **9**, **12**, **15** and **26d** showed moderate antioxidant activity, whereas the compounds **26e** and **26g** displayed weak nitric oxide scavenging properties compared to the control. Compounds that can neutralize the effects of NO formation may be of interest for avoiding the severe effects of extreme NO generation in living organisms. This study shows that the compounds are potent nitric oxide scavengers. Nitric oxide generated from sodium nitroprusside reacts with oxygen to form nitrite, therefore, these compounds may inhibit nitrite formation by competing with oxygen and reacting with nitric oxide. Nitric oxide is a gassy signaling molecule known to have an important function in a variety of biological systems. Nitric oxide free radicals are formed non-enzymatically and have a short lifetime, but cause harm to most biomolecules, including proteins and DNA. Nitric oxide has a role in many of human diseases such as inflammation and tumors.

## 3. Experimental Section

### 3.1. General Information

All melting points were determined on an Electrothermal apparatus and are uncorrected. IR spectra were recorded (KBr discs) on a Shimadzu FT-IR 8201 PC spectrophotometer. ^1^H-NMR spectra were recorded in CDCl_3_ and (CD_3_)_2_SO solutions on a Varian Gemini 300 MHz spectrometer and chemical shifts are expressed in δ units using TMS as an internal reference. Mass spectra were recorded on a Shimadzu GC-MS QP 1000 EX instrument. Elemental analyses were carried out at the Microanalytical Canter of Cairo University. The starting reagents **3** [41], **5** [47], **6** [48], **11a** [49], **11b** [50], **11c** [51], **11d** [52], **17** [53], **22** [54] and **27** [42,43,44,45,46] were prepared as previously described in the respective references.

#### 3.1.1. Synthesis of 2-Hydroxy-*N*-phenylbenzohydrazonoyl Bromide (**4**)

To a solution of *N*-bromosuccinimide (10 mmol) dissolved in CH_2_Cl_2_ (70 mL) at 0 °C was added dimethyl sulfide (1.12 g, 18 mmol) and the reaction mixture was stirred for 15 min at 0 °C and then kept in a deep freezer (−20 °C) overnight. To this solution was added the hydrazone (9 mmol) in CH_2_Cl_2_ (20 mL) and the reaction mixture was left in the deep freezer overnight and then allowed to warm to room temperature for 2 h. The progress of the reaction was monitored by TLC. The reaction mixture was quenched with cold water. The contents were extracted with CH_2_Cl_2_ (40 mL), washed with water (2 × 40 mL) and dried (MgSO_4_). The solvent was removed under reduced pressure and crystallization from MeOH afforded the final product 2-hydroxy-*N*-phenylbenzohydrazonoyl bromide (**4**). Green solid; yield (86%); mp 175 °C; IR: 3433 (OH), 3317 (NH) and 1623 (C=N) cm^−1^. ^1^H-NMR (DMSO-*d*_6_): 6.94–8.17 (m, 9H, ArH), 10.25 (s, H, NH) and 10.46 (s, 1H, OH) ppm. MS *m/z* (%) = 291 (M^+^, 72); Anal. Found for C_13_H_11_BrN_2_O (291.14): C, 53.68; H, 3.84; N, 9.57; requires: C, 53.63; H, 3.81; N, 9.62%.

#### 3.1.2. Synthesis of 2-(5-(2-Hydroxyphenyl)-2,4-diphenyl-2H-1,2,4-triazol-3(4*H*)-ylidene)malononitrile (**9**) and 2-(3-(2-Naphthoyl-1,4-diphenyl-1*H*-1,2,4-triazol-5(4*H*)-ylidene)malononitrile (**10**)

To a mixture of 2-[(methylthio)-(phenylamino)methylene]malononitrile (**5**, 1.075 g, 0.005 mol) and hydrazonoyl chloride **4** or **6** (0.005 mol) in ethanol (20 mL) was added triethylamine (0.7 mL, 0.005 mol) and the mixture was refluxed till methane thiol ceased to evolve (6 h). The precipitate that was formed was filtered off and crystallized from DMF/MeOH mixture to give the 1,2,4-triazole derivatives **9** and **10**, respectively. The physical constants of the compounds prepared are listed below.

*2-(5-(2-Hydroxyphenyl)-2,4-diphenyl-2H-1,2,4-triazol-3(4H)-ylidene)malononitrile* (**9**). Pale green solid (71%), mp 171 °C. IR (KBr) ν 3433 (OH) and 2258, 2206 (CN) cm^−1^; ^1^H-NMR (DMSO-*d*_6_): 7.01–8.32 (m, 14H, ArH) and 10.45 (s, 1H, OH) ppm. MS *m/z* (%) 377 (M^+^, 18), Anal. Found: C, 73.16; H, 3.98; N, 18.64%; C_23_H_15_N_5_O (377.13) requires: C, 73.20; H, 4.01; N, 18.56.

*1,4-Diphenyl-3-naphthoyl-5-dicyanomethylene-1,2,4-triazole carboxylate* (**10**). Yellow solid (50%), mp 288 °C. IR (KBr) ν 2199, 2188 (CN) and 1690 (C=O) cm^−1^; ^1^H-NMR (DMSO-*d*_6_): 7.32–8.04 (m, 17H, ArH); MS *m/z* (%) 439 (M^+^, 10), Anal. Found: C, 76.49; H, 3.95; N, 15.91; C_28_H_17_N_5_O (439.47) requires: C, 76.52; H, 3.90; N, 15.94%.

#### 3.1.3. General Procedure for the Preparation of Bis([1,2,4]triazolo[4,3-*a*]pyrimidine) Derivatives **12**,**13**), Bis([1,2,4]triazolo[4,5-*a*]benzimidazole) Derivative **14** and 3-(2-Hydroxyphenyl)-8-methyl-1-phenyl-1*H*-pyrimido[1,2-*b*][1,2,4,5]tetrazine-6-(4*H*)-one (**15**)

A mixture of the thioxo or 2-methylthio derivatives **11a**–**d** (10 mmol) and 2-hydroxy-*N*-phenylbenzohydrazonoyl bromide (**4**, 1.455 g, 5 mmol) were refluxed in chloroform (20 mL) in the presence of triethylamine (1.01 g, 1.39 mL, 10 mmol) till hydrogen sulfide or methane thiol ceased to evolve (4–6 h) and then cooled. The cold reaction mixture was then poured onto cold petroleum ether with stirring. The solid that precipitated was collected, washed with water several times, dried and recrystallized from MeOH to give the final products **12**–**15**.

*Ethyl-1,5-dihydro-3-(2-hydroxyphenyl)-7-methyl-1,5-diphenyl-[1,2,4]triazolo[4,3-a]pyrimidine-6-crboxylate* (**12**). Pale yellow solid (82%), mp 206 °C. IR (KBr) ν 3425 (OH) and 1666 (C=O) cm^−1^; ^1^H-NMR (DMSO-*d*_6_): 1.23 (t, 3H, OCH_2_CH_3_), 2.41 (s, 3H, CH_3_), 4.14 (q, 2H, OCH_2_CH_3_); 5.37 (s,1H, pyrimidine-1H); 6.99–8.12 (m, 14H, ArH) ppm; 10.44 (s, 1H, OH); MS *m/z* (%) 452 (M^+^, 17), Anal. Found: C, 71.69; H, 5.29; N, 12.42; C_27_H_24_N_4_O_3_ (452.5) requires: C, 71.67; H, 5.35; N, 12.38%.

*3-(2-Hydroxyphenyl)-7-methyl-1-phenyl-[1,2,4]triazolo[4,3-a]pyrimidin-5(1H)-one* (**13**). Yellow brown solid (74%), mp 184 °C. IR (KBr) ν 3433 (OH), and 1680 (C=O) cm^−1^; ^1^H-NMR (DMSO-*d*_6_): 2.35 (s, 3H, CH_3_), 6.16 (s, 1H, pyrimidine-H), 6.97–8.02 (m, 9H, ArH), 10.41 (s, 1H, OH); MS *m/z* (%) 318 (M^+^, 31), Anal. Found: C, 67.89; H, 4.48; N, 17.57; C_18_H_14_N_4_O_2_ (319.12) requires: C, 67.91; H, 4.43; N, 17.60%.

*3-(2-Hydroxyphenyl)-1-phenyl-1,2,4]triazolo[4,5-a]benzimidazole* (**14**). Brown solid (80%), mp 163 °C. IR (KBr) ν 3425 (OH) and 1605 (C=N) cm^−1^; ^1^H-NMR (DMSO-*d*_6_): 6.97–8.34 (m, 13H, ArH) and 10.48 (s, 1H, OH) ppm; MS *m/z* (%) 326 (M^+^, 25), Anal. Found: C, 73.58; H, 4.38; N, 17.23; C_20_H_14_N_4_O (326.35) requires: C, 73.61; H, 4.32; N, 17.17%.

*3-(2-Hydroxyphenyl)-8-methyl-1-phenyl-1H-pyrimido[1,2-b][1,2,4]tetrazine-6(4H)-one* (**15**). Pale green solid (63%), mp 170 °C. IR (KBr) ν 3433 (OH), 3317 (NH), and 1690 (C=O) cm^−1^; ^1^H-NMR (DMSO-*d*_6_): 6.93–8.13 (m, 13H, Ar’s), 9.51 (s, 1H, NH) and 10.48 (s, 1H, OH); MS m/z (%) 333 (M^+^, 32), Anal. Found: C, 64.81; H, 4.49; N, 21.06; C_18_H_15_N_5_O_2_ (333.34) requires: C, 64.86; H, 4.54; N, 21.01%.

#### 3.1.4. Synthesis of 2-(4,5-Dihydro-5-imino-4-phenyl-1,3,4-thiadiazol-2-yl)phenol (**20**)

A mixture of 2-hydroxy-*N*-phenylbenzohydrazonoyl bromide (**4**, 2.90 g, 10 mmol) and the appropriate of potassium thiocynate (**16**) in EtOH (20 mL) was boiled under reflux for 6 h. The cold reaction mixture was then poured onto ice-cold hydrochloric acid with stirring. The solid that precipitated was collected. The resulting solids filtered, washed with water several times and crystallized from MeOH to give the final product *2-(4,5-dihydro-5-imino-4-phenyl-1,3,4-thiadiazol-2-yl)phenol* (**20**). Yellow brown solid (74%) mp 176 °C. IR (KBr) ν 3433 (OH) and 3115 (NH) cm^−1^; ^1^H-NMR (DMSO-*d*_6_): 7.03–8.69 (m, 10H, ArH and =NH) and 10.49 (s, 1H, OH); MS *m/z* (%) 269 (M^+^, 16), Anal. Found: C, 62.39; H, 4.14; N, 15.63; C_14_H_11_N_3_OS (269.32) requires: C, 62.43; H, 4.12; N, 15.60%.

#### 3.1.5. Synthesis of 1-(2-Hydroxyphenyl-3-phenyl-1,3,4-thiadiazol-4,(3*H*)-ylidene)hydrazine (**21**) and 4,5-Dihydro-4-phenyl-5-(phenylimino)-1,3,4-thiadiazol-2-yl)phenol (**24**)

A mixture of 2-hydroxy-*N*-phenylbenzohydrazonoyl bromide (**4**, 2.90 g, 10 mmol) and methyl hydrazinecarbodithioate (**17**, 10 mmol) or methyl hydrogenphenylcarbonimidodithioate (**22**, 1.83 g, 10 mmol) in EtOH (20 mL) was stirred in the presence of triethylamine (1.01 g, 1.39 mL, 10 mmol). The resulting mixture was refluxed for 4 h, and then cooled. The cold reaction mixture was then poured onto ice-cold water with stirring. The solid that precipitated was collected. The resulting solids were filtered, washed with water several times and recrystallized from MeOH to give the final products **21** and **24**.

*1-(2-Hydroxyphenyl-3-phenyl-1,3,4-thiadiazol-4,(3H)-ylidene)hydrazine* (**21**). Green solid (69%) mp 185 °C. IR (KBr) ν 3433 (OH) and 3190, 3171 (NH_2_) cm^−1^; ^1^H-NMR (DMSO-*d*_6_): 5.83 (s, 2H, NH_2_), 7.32–8.04 (m, 11H, ArH) and 10.43 (s, 1H, OH); MS *m/z* (%) 284 (M^+^, 29), Anal. Found: C, 59.23; H, 4.21; N, 19.64; C_14_H_12_N_4_OS (284.34) requires: C, 59.14; H, 4.25; N, 19.70%.

*4,5-Dihydro-4-phenyl-5-(phenylimino)-1,3,4-thiadiazol-2-yl)phenol* (**24**). Pale green solid (75%) mp 150 °C. IR (KBr) ν 3379 (OH) and 1617 (C=N) cm^−1^; ^1^H-NMR (DMSO-*d*_6_): 7.01–8.15 (m, 14H, ArH) and 10.41 (s, 1H, OH); MS *m/z* (%) 345 (M^+^, 41), Anal. Found: C, 69.58; H, 4.34; N, 12.14; C_20_H_15_N_3_OS (345.42) requires: C, 69.54; H, 4.38; N, 12.17%.

### 3.2. Biology

#### 3.2.1. Antibacterial Activities

Bacterial strains utilized in the present work were obtained from the American Type Culture Collection (ATCC; Manassas, VA, USA). The antimicrobial evaluation of triazoles, tetrazine and thiadiazoles compounds were carried out via the cup diffusion procedure [55]. The antibacterial activity was tested against the Gram-positive bacterial strains *Staphylococcus aureus* (*S. aureus*) ATCC 25923 and *Bacillus megaterium* (*B. megaterium*) ATCC 14591 and the Gram-negative ones *Escherichia coli* (*E. coli*) ATCC 25922 and *Pseudomonas aeruginosa* (*P. aeruginosa*) ATCC 27853. The studied compounds were dissolved in dimethylsulfoxide (DMSO) at a concentration 1 mg/mL. The Luria-Bertani Agar (LBA) Medium (5 g yeast extract, 10 g bacto-tryptone, 10 g NaCl, and 20 g agar in 1 Liter de-ionized water) was used for inoculation and bacterial growth. An aliquot of the solution of the compounds equivalent to 100 μg was placed separately in cups cut in the agar. The LBA plates were incubated at 37 °C for 24 h and the resulting inhibition zones were measured. From the inhibition zone diameter data analysis, the antimicrobial activities of compounds against Gram-positive and Gram-negative bacteria were estimated.

#### 3.2.2. Minimum Inhibitory Concentrations (MIC)

Determination of MIC of compounds was completed according to the standard method summarized by the CLSI/NCCLS norms [56]. In brief, to sterilized LB broth solution, several concentrations (0.5–50 μg/mL) of the compounds in DMSO were added individually. The test cultures of Gram-negative *E. coli*, *P. aeruginosa* and Gram-positive *S. aureus*, *B. megaterium* bacterial strains were selected for the present study. All bacterial strains were grown in LB broth separately with 50 μL LB containing approximately 5 × 10^4^ colony forming units (CFU) of 18 h grown cultures of each microorganism to be tested. Microorganisms were inoculated and the final optical density (OD) of the test solution was kept as ≈0.5 and the compounds were incubated for 24 h at 37 °C. Respective blanks (culture and broth alone) were conserved, accordingly. Following incubation, the turbidity of the growth medium was measured at 600 nm. The concentration at which the solution (OD is 0.5) turns turbid, was measured as the MIC of the test samples. Experiments were carried out in duplicate.

#### 3.2.3. DNA Cleavage Assay

The DNA cleavage study of the compounds was performed as described by Youssef *et al.* [57]. In brief, the compounds were dissolved in DMSO (0.5 mg/mL) and 4 μg was added to 2 µg of *E. coli* DNA. DNA only and DNA with DMSO were used as a control. The reactions were done for one hour at 37 °C. A solution of 10% w/v ficol 400, 0.06% w/v bromophenol blue, and 0.5% w/v SDS was added to the reaction mixtures before running the horizontal gel. The DNA was assessed via agarose gel electrophoresis done using 1.0% agarose gel at a constant voltage of 80 V for 60 min in TEA (40 mM Tris-HCl pH 7.9, 5 mM sodium acetate, 1 mM EDTA) buffer. The agarose gels were stained with ethidium bromide (0.5 µg/mL) and visualized through a UV transilluminator and photographed using a digital camera.

#### 3.2.4. Superoxide Dismutase (SOD) Mimetic Catalytic Activity Assay

The SOD mimetic catalytic activity of compounds was evaluated by nitroblue tetrazolium/phenazine methosulphate/reduced nicotinamide (NBT/PMS/NADH) to photogenerate a reproducible and continuous flux of O_2_^•−^ at pH = 8.3 (phosphate buffer). Reduction of NBT to blue formazan was employed as an indicator of O_2_^•−^ production and observed spectrophotometrically at 560 nm. PMS (0.93 mM) was added to an aqueous solution of NBT (0.03 mM), NADH (0.47 mM), and phosphate buffer (final volume of 2 mL). The reaction in blank samples and in the presence of compounds was measured for 5 min. For comparison purposes, the activity of native horseradish superoxide dismutase (HR SOD) was also measured. The percent of inhibition of free radical production from SOD mimetic catalytic activity was considered by the following equation:

$$I\%=[(\Delta A_{control}-\Delta A_{sample})/\Delta A_{control}]\times 100$$

where A_control_ is the absorbance of the control reaction (containing all reagents except the test compound) and A_sample_ is the absorbance of the test compound. Tests were carried at in triplicate.

#### 3.2.5. 2,2,-Diphenyl-1-Picrylhydrazyl (DPPH) Free Radical Scavenging Activity Assay

The electron donation ability of compounds was evaluated from the reduction of a purple-colored stable free radical DPPH into the yellow-colored diphenylpicryl hydrazine. When odd electrons in DPPH^•^ are removed the absorbance at 517 nm is reduced steadily due to the increase of non-radical DPPH forms. As this electron becomes paired off in the presence of a free radical scavenger, the absorption disappears and the resulting decolorization is stoichiometric with respect to the number of electrons taken up. Briefly, 1 mL of compounds (1 μg) in DMSO/ ethanol (1:1 v:v) or standard (vitamin C) were added to 4 mL of 0.004% (w/v) ethanol solution of DPPH and vortexes thoroughly. After a 30 min incubation period at 30 °C, the absorbance was read against blank (DMSO/methanol 1:1 *v:v*) at 517 nm. The percent of inhibition of free radical production from DPPH was considered by the following equation:

$$I\%=[(A_{control}-A_{sample})/\;A_{control}]\times 100$$

where A_control_ is the absorbance of the control reaction (containing all reagents except the test compound) and A_sample_ is the absorbance of the test compound. Tests were carried out in triplicate.

#### 3.2.6. 2,2'-Azino-bis-3-ethylbenzthiazoline-6-sulphonic Acid (ABTS) Radical Cation Assay

ABTS forms a relatively steady free radical, which decolorizes to its non-radical form. The analysis of ABTS free radical scavenging activity was assessed spectrophotometrically according to the method reported in [58]*.* A mixture of ABTS (1 mL, 0.1 g/100 mL) and MnO_2_ (1.5 mL, 25 mg/mL), prepared in phosphate buffer (PB: pH 7.4, 100 mM( was vortexed, centrifuged and decanted. Prior to the evaluation, the resulting green-blue solution was diluted in PB to give an absorbance (A_control_ of ABTS radical solution) at 734 nm of 0.600 in a 1 cm quartz cuvette and equilibrated to 30 °C, the temperature at which all the estimates were done. Antioxidant activities of the verified compounds were assessed using the radical cations of ABTS. The absorbance (A_sample_) was determined upon the addition of 2 g of the compounds in DMSO/PB (1:1 v/v) to the ABTS mixture. Ascorbic acid was used as a standard antioxidant (positive control). Blank samples were run by solvent without ABTS. The decrease in absorbance is expressed as % inhibition which is calculated from the following formula:

$$(\%)\;Ihibition=[(A_{control}-A_{sample})/A_{control}]\times 100$$

where A_control_ is the absorbance of the control reaction (containing all reagents except the test compound) and A_sample_ is the absorbance of the test compound. All total antioxidant activity data are the average of triplicate analyses.

#### 3.2.7. Nitric Oxide (NO) Scavenging Assay

The nitric oxide scavenging activity procedure was carried out according to [59], whereby sodium nitroprusside generates NO radicals, which interact with oxygen to produce nitrite ions. These can be assessed using the Greiss reagent (0.1% *N*-(1-naphthyl)ethylenediamine dihydrochloride, 2% H_3_PO_4_ and 1% sulfanilamide). Scavengers of nitric oxide participate with oxygen leading to less production of nitrite ions. For the assessment, sodium nitroprusside (10 mM) in PB saline was mixed with compounds (2 μg) and standard. The reaction mixtures were incubated at 25 °C for 150 min and after that, 0.5 mL of Griess reagent [60] was added. The absorbance of the pink colour formed during diazotization of nitrite with sulphanilamide and subsequent coupling with naphthylethylenediamine was read at 546 nm and referred to the absorbance of standard solutions of sodium nitrite treated in the same way with Griess reagent. Nitric oxide scavenging activity was calculated by the following equation:

$$\%\;Nitric\;oxide\;scavenging\;activity=[(Acontrol-A_{sample})/A_{control}]\times 100$$

where A_control_ is the absorbance of the control reaction (containing all reagents except the test compound) and A_sample_is the absorbance of the test compound. All total antioxidant activity data are the average of triplicate analyses.

## 4. Conclusions

In conclusion, a facile synthesis of novel bioactive heterocycles such as triazoles, tetrazine and thiadiazoles is reported. The compounds were evaluated for their biological properties and gratifyingly, the compounds **5**, **24** and **26h** exhibited excellent antibacterial and antioxidant activities. These compounds also effectively promoted the cleavage of genomic DNA in the absence of external additives, which suggests a potential application of these compounds as chemotherapeutic agents in anticancer treatment.
